# Multispacer Sequence Typing for *Mycobacterium tuberculosis* Genotyping

**DOI:** 10.1371/journal.pone.0002433

**Published:** 2008-06-18

**Authors:** Zoheira Djelouadji, Catherine Arnold, Saheer Gharbia, Didier Raoult, Michel Drancourt

**Affiliations:** 1 Unité des Rickettsies CNRS UMR6236, IFR 48, Faculté de Médecine, Université de la Méditerranée, Marseille, France; 2 Applied and Functional Genomics Unit, Centre for Infections, Health Protection Agency, London, United Kingdom; Centre for DNA Fingerprinting and Diagnostics, India

## Abstract

**Background:**

Genotyping methods developed to survey the transmission dynamics of *Mycobacterium tuberculosis* currently rely on the interpretation of restriction and amplification profiles. Multispacer sequence typing (MST) genotyping is based on the sequencing of several intergenic regions selected after complete genome sequence analysis. It has been applied to various pathogens, but not to *M. tuberculosis*.

**Methods and Findings:**

In *M. tuberculosis*, the MST approach yielded eight variable intergenic spacers which included four previously described variable number tandem repeat loci, one single nucleotide polymorphism locus and three newly evaluated spacers. Spacer sequence stability was evaluated by serial subculture. The eight spacers were sequenced in a collection of 101 *M. tuberculosis* strains from five phylogeographical lineages, and yielded 29 genetic events including 13 tandem repeat number variations (44.82%), 11 single nucleotide mutations (37.93%) and 5 deletions (17.24%). These 29 genetic events yielded 32 spacer alleles or spacer-types (ST) with an index of discrimination of 0.95. The distribution of *M. tuberculosis* isolates into ST profiles correlated with their assignment into phylogeographical lineages. Blind comparison of a further 93 *M. tuberculosis* strains by MST and restriction fragment length polymorphism-IS*6110* fingerprinting and mycobacterial interspersed repetitive units typing, yielded an index of discrimination of 0.961 and 0.992, respectively. MST yielded 41 different profiles delineating 16 related groups and proved to be more discriminatory than IS*6110*-based typing for isolates containing <8 IS*6110* copies (*P*<0.0003). MST was successfully applied to 7/10 clinical specimens exhibiting a Cts ≤ 42 cycles in internal transcribed spacer-real time PCR.

**Conclusions:**

These results support MST as an alternative, sequencing-based method for genotyping low IS*6110* copy-number *M. tuberculosis* strains. The *M. tuberculosis* MST database is freely available (http://ifr48.timone.univ-mrs.fr/MST_MTuberculosis/mst).

## Introduction

*Mycobacterium tuberculosis* is a successful worldwide human pathogen responsible for 2–3 million deaths and 8–10 million new cases per year [1], [2], most of them being in resource poor countries. Genotyping of *M. tuberculosis* is useful for population dynamics analysis as well as the identification of outbreaks [3]. Genotyping is based upon genomic variability in *M. tuberculosis*, and, using a combination of two alleles at *katG463* and *gyrA95,* the species can be broadly divided into three major genetic groups [4]. Fingerprinting techniques based on repetitive DNA sequences can further differentiate these groups into genetic families including the East African Indian, Beijing, Haarlem and X, and Latin American and Mediterranean families [5]–[7]. Spoligotyping studies delineated nine major clades including genotypes responsible for major outbreaks [8]–[11], which were supported by a study analysing neutral variation found within genes associated with drug resistance [12]. Deletion analysis shed further light on the deeper structure of the *M. tuberculosis* complex and found six main lineages and 15 sublineages of *M. tuberculosis* [13]. Although the genetic markers used in these studies were different, the overall phylogenetic structure of the species was the same between the different methods and demonstrated that *M. tuberculosis* was clonal. Genotyping methods currently rely upon analysis of restriction profiles including pulsed-field gel electrophoresis (PFGE) [14], [15], restriction fragment length polymorphisms (RFLP) using IS*6110* probing [16], amplification profiles of selected regions of variable number tandem repeat (VNTR) including the exact tandem repeat (ETR) regions [17] and mycobacterial interspersed repetitive units (MIRU) [18], spoligotyping [19] and deletion and insertion site mapping [20]. Recently, single nucleotide polymorphism (SNP) analysis including SNP located in intergenic spacers was performed, delineating either six [9] or nine broad groups [21]. However, systematic sequencing of intergenic spacers has not been done for *M. tuberculosis* genotyping.

We investigated Multispacer Sequence Typing (MST) for *M. tuberculosis* genotyping. This technique is based on a single sequence analysis of several intergenic regions selected by complete genome sequence comparison, resulting in a sequencing-based, genotypic profile [22]. MST has been previously used to genotype several pathogens otherwise demonstrated to be highly homogenous, including *Yersinia pestis* [22], *Bartonella quintana* [23], *Rickettsia conorii* [24], *Rickettsia prowazekii* [25], *Coxiella burnetii* [26] and *Bartonella henselae* [27]. Intergenic spacers have been investigated for the identification of *Mycobacterim tuberculosis* complex species [28], but has never been applied to *M. tuberculosis* genotyping. We herein developed a sequencing-based approach for the genotyping of *M. tuberculosis* isolates from our laboratory and further compared MST with a blinded panel of 93 IS*6110*-RFLP and MIRU/VNTR-characterised strains.

## Methods

### Identification and selection of spacers for MST

The genome sequences of *M. tuberculosis* strains H37Rv (GenBank: AL123456) [29] and CDC1551 (GenBank: AE000516) [30] were analysed using the EMBOSS software (http://www.emboss.sourceforge.net). Spacer sequences were extracted from both genomes using perl script software. Homologous spacer sequences were compared by using Difseq software in EMBOSS. NCBI Blast was then used to visualise differences between homologous spacer sequences. Spacers fulfilling the following criteria were retained: 1) sequence length of ≤500-bp so that experimental sequences would be in the sequencing range of current automatic sequencers, 2) software script-filtered range of sequence similarity between both *M. tuberculosis* genomes of 70–99%; the 70% cut-off was chosen to ensure that comparison included two homologous spacers and excluded two unrelated genomic regions; the 99% cut-off was chosen to ensure a minimum variability in spacer sequence, 3) a difference between *M. tuberculosis* H37Rv and CDC1551 sequence of >4-bp. Spacer homology between the two *M. tuberculosis* genomes was further ensured by the presence of homologous genes upstream and downstream of the spacer sequence. A dot plot was constructed for each spacer in order to visualise the type of genetic events responsible for spacer sequence heterogeneity, i.e. tandem repeat, mutation, insertion or deletion. PCR primers were designed for each spacer using the Primer3 software program (INFOBIOGEN, Evry, France).

### Bacterial isolates

Initial development of MST for *M. tuberculosis* genotyping, was carried out by amplification and sequencing of spacers of a sample of 100 strains isolated in our laboratory in 2001–2005, in addition to reference strain H37Rv CIP 64.31 purchased from the Collection Institut Pasteur (CIP, Paris, France). The laboratory covers an area with over two million inhabitants with a significant migrant population. Strains were identified as *M. tuberculosis* on the basis of conventional biochemical test results [31] and ITS-probing (GenProbe, San Diego, CA). *M. tuberculosis* isolates were classified into phylogeographical lineages using the molecular scheme previously developed by Gagneux and collaborators [13]. This study has been approved by the local ethic committee, Marseilles. For each isolate, a single colony grown on 5% sheep blood agar (Biotechnology Appliquée, Dinan, France) was taken using a sterile loop, mixed with freezing beads for storage at −20°C prior to inactivation as previously described [32] and DNA extraction using Qiagen kit (Qiagen, Courtaboeuf, France).

To further compare MST with reference IS*6110*-RFLP genotyping, we included the DNA extracted using the QIAmp DNA minikit (Qiagen, Crawley, United Kingdom) from 93 *M. tuberculosis* isolates from the United Kingdom [33]. The isolates have been previously characterised as belonging to major genetic group (MGG1) (45; 48.38%), MGG2 (39; 41.93%) and MGG3 (9; 9.67%) by analysing nucleotide polymorphisms in the catalase-peroxidase and gyrase A subunit gene sequences [4]. IS*6110-*RFLP analysis was performed [16], IS*6110* clusters were further resolved using VNTR/MIRU as second line [34]. Seventy-two different IS*6110-*RFLP profiles were generated from 93 *M. tuberculosis* isolates including 58 unique profiles and 14 profiles of 2–6 isolates epidemiologically clustered isolates totalling 35 *M. tuberculosis* isolates. One epidemiological cluster contained four isolates, two clusters comprised three isolates each and four clusters comprised two isolates. Two replicates of five isolates were also included in the study. 75/93 isolates were unique by at least one of the three methods used.

### Clinical specimens

We selected 10 respiratory tract clinical specimens from our laboratory, which cultured *M. tuberculosis* identified on the basis of conventional biochemical test results [30]. All specimens also yielded a positive internal transcribed spacer (ITS) amplification with Cts ranging from 21 to 45 cycles using real time PCR [35]. DNA extraction as well as PCRs and sequencing of the eight spacers were performed as described above. Negative controls consisted of respiratory tract specimens which remained negative for *M. tuberculosis* by both culture and ITS probing.

### MST analysis and comparison with IS*6110*-RFLP and MIRU/VNTR

MST PCRs were carried out in a final volume of 50 µl containing 33 µl H_2_O, 5 µl 10× buffer (Qiagen), 2 µl 25× MgCl_2_, 5 µl 10× dDNTP, 1 µl forward primer (10 pmol/µl), 1 µl reverse primer (10 pmol/µl), 0.25 µl hotstart Taq (Qiagen) and 2 µl target DNA. Appropriate negative controls consisting of PCR mix without target DNA were also included. PCRs were performed according to the following program: 15 min enzyme activation at 95°C, followed by 34 cycles consisting of 95°C for 30 s, 60°C for 30 s, 72°C for 1 min and 5 min final elongation step at 72°C. After visualising the size of amplified fragments by agarose gel electrophoresis, purified PCR products were sequenced using the BigDye Terminator v1.1 Cycle Sequencing kit (Applied Biosystems). Sequencing electrophoresis was performed by 3100 genetic analyser (Applied Biosystems) in both directions. The sequences were edited using the Auto assembler program (Applied Biosystems), aligned using CLUSTAL W (http://bioinfo.hku.hk/services/analyseq/cgi-bin/clustalw_in.pl), and NPS Multalin multiple alignment (http://npsa-pbil.ibcp.fr). Direct visual examination of edited alignment was also carried out to minimize the risk of alignment error. The sequences were then compared with a local database of *M. tuberculosis* spacer sequences. Blinded comparison of MST and IS*6110*-RFLP was carried out with MST analyses performed in Marseille as described above using coded *M. tuberculosis* DNAs extracted in London.

### Reproducibility, discriminatory power and statistical analysis

We evaluated the stability of MST typing applied to serial *M. tuberculosis* isolates, isolated from five different patients at different intervals of time and also with four *M. tuberculosis* isolates sub-cultured twice. The difference between phylogeographical clustering [13] and MST clustering of *M. tuberculosis* isolates was tested by using the Chi Square test (Epi Info version 3.4.1, Centers for Diseases Control and Prevention, Atlanta, USA).

The discrimination power of the MST approach and IS*6110*-RFLP method was calculated using the Hunter Gaston Index, which was estimated as
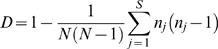
where N was the total number of isolates in the sample population, S was the total number of types described, and n_j_ is the number of isolates belonging to the j^th^ type [36].

## Results

### Selection of spacers for typing and analysis by MST

Comparison of *M. tuberculosis* H37Rv and CDC1551 strain genome sequences [29], [30] showed 83 spacers of ≤500-bp exhibiting 70–99% sequence similarity between both genomes of which only 14 spacers exhibited >4-bp differences between both *M. tuberculosis* genomes according to the criteria outlined below. Primer sequences derived from these 14 spacers are shown in Table 1. When initially applied to a limited collection of 20 *M. tuberculosis* isolates, PCR negative controls remained negative and 6/14 spacers: MST5, MST6, MST7, MST9, MST10, and MST14 exhibited only one or two genotypes with 17/20 (85%) isolates belonging to the same genotype. As these six spacers demonstrated limited variability they were excluded from further study. In contrast, eight spacers were found to be highly variable among the 20 isolates (Table 2). These eight spacers comprised the four previously described Exact Tandem Repeat ETR-B (MST4), ETR-C (MST11), ETR-D alias MIRU4 (MST12) [17], [37] and Mtub21 (MST13) [38]. One spacer herein designed as MST8 had been previously shown to contain one SNP designated MT2221 [21]. Three spacers MST1, MST2 and MST3 were newly identified by our analysis.

**Table 1:** Primers used for PCR amplification and sequencing of *M. tuberculosis* isolates.

Spacer	ORF (Upstream and downstream spacer sequence)	Nucleotide sequence (5’-3’) and position*	PCR product size* (bp)
MST1	Conserved hypothetical protein	71487 GCTGGCCGATCTGCGCGC	308
	Conserved hypothetical protein	71795 GATGGTCTCCCGGCTGAT	
MST2	Mce family protein MCEF1	206690 GCCCGGCCAGCGGTGAACTGG	338
	Mce associated membrane protein	207028 GCGCCAAGGCCACCGGCCAA	
MST3	Conserved hypothetical protein	1622918 TCGAGGATTCTGGGACTAT	275
	Conserved hypothetical protein	1623193 CTGTGGCAGGCTCCCGGTAG	
MST4	Ubiquinol-cytochrome C reductase	2461301 ATGGGTTCGCCAGACGGCGAG	305
(ETR-B)	Conserved trans-membrane protein	2461606 GATCAGCTACGGGTTGGCCG	
MST5	Transcriptional regulatory protein	3423121 CGGCCAGCCGCGGCGGACGA	291
	Acyl coA Deshydrogenase FADE22	3423412 GATTGCGTTGCTGGACGGCCC	
MST6	Helicase hely ATP-dependant DNA	2351811 CTGTGAGATCGGTGTGCTTT	603
	Sec-independent protein translocase	2352414 CTGCTGATCGTGATGCTGAA	
MST7	Conserved hypothetical protein	2401532 GCACCGGATTCAACGTATTC	507
	Conserved hypothetical protein	2402039 TAGTAGGGCACTAGCACCTC	
MST8	PE-PGRS family protein	2424819 GCCGCAATCACAAACGACAT	455
(MT2221)	Penicillin-binding membrane protein	2425274 GCTACTTCGACGACGTGTAT	
MST9	Conserved integral membrane protein	2990382 CTTCATGACGTTGGATCGCT	525
	Conserved hypothetical protein	2990907 CGCAATGCGACTCGAATTTC	
MST10	RNA polymerase sigma factor	3023146 CTTTGGGCGATTTCATCGAG	481
	Iron-dependent repressor-activator	3023627 CTCTTCCTCGAGGTCGTAGAT	
MST11	Conserved hypothetical protein	577151 AGGTGTTAGAGGTGGTGGAT	692
(ETR-C)	Conserved integral membrane protein	577843 AACCAAAGTCATATTGGGATGA	
MST12	Putative histidine kinase Senx3	580482 GTTGATCGAGGCCTATCACG	637
(ETR-D)	Sensory transduction protein Regx3	581119 GAATAGGGCTTGGTCACGTA	
MST13	Conserved hypothetical protein	1955383 CGAGTTCACCGTCCATCATC	554
(Mtub21)	Possible penicillin-binding protein	1955937 GAGACAACGGTCATCGACTT	
MST14	Transcriptional regulatory protein	3595692 CATAGTGAGGAGTAACGACTA	648
MST14	Component sensor kinase	3596340 CTGATCATGGTGATCACCGA	

* with reference to *M. tuberculosis* H37Rv genome sequence (GenBank: AL123456).

**Table 2:** Polymorphism characteristics of 8 variable intergenic spacers studied in *M. tuberculosis* isolates.

Spacer	No. of alleles	No and size of repeat units (bp)	No and position of SNP*	Size of deletion (bp)
MST13 (Mtub21)	6	4×57	A1C	23
MST4 (ETR-B)	5	5×57	-	-
MST12 (ETR-D)	4	5×77	T45G	24
MST11 (ETR-C)	4	3×58	T1C	24
MST8 (MT2221)	3		A26G, A57C, A259C	
MST1	4	-	A20G, T103C	37
MST2	3	-	C294G	57
MST3	3	-	T148A, T210G	-

* the number between the locus shows the position of the variable nucleotide, the locus before the number is the variable nucleotide within specific genotype, and the locus after the number is the nucleotide in other isolates.

### *M. tuberculosis* strains

**Figure 1:**
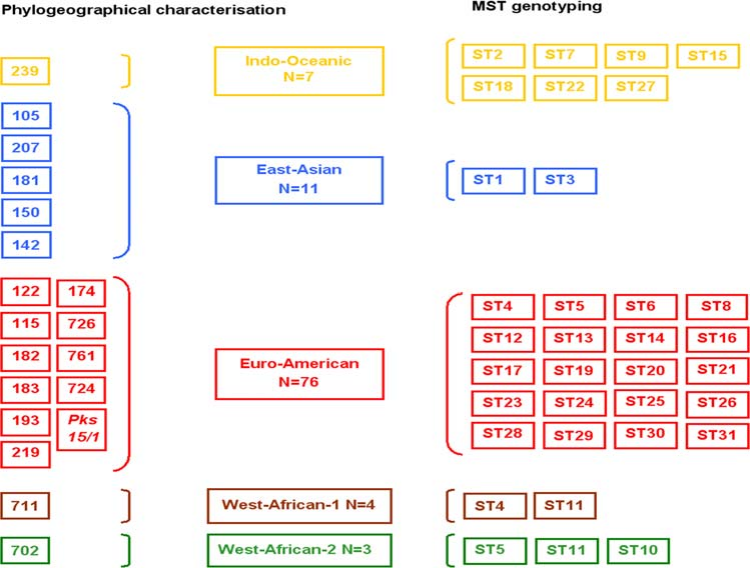
Comparison of the distribution of 101 *M. tuberculosis* into five phylogeographical lineages and MST profiles defined in present study. From left to right, large polymorphism sequences, phylogeographical lineages defined by Gagneux et al [13], spacer types obtained by MST.

The 100 *M. tuberculosis* clinical isolates and H37Rv reference strain were distributed into five phylogeographical lineages. Seven isolates were classified into the Indo-Oceanic lineage, 11 isolates into the East-Asian lineage, 76 isolates into the Euro-American lineage, four isolates into the West-African lineage 1 and three isolates into the West-African lineage 2 (Figure 1).

### *M. tuberculosis* MST database

**Figure 2:**
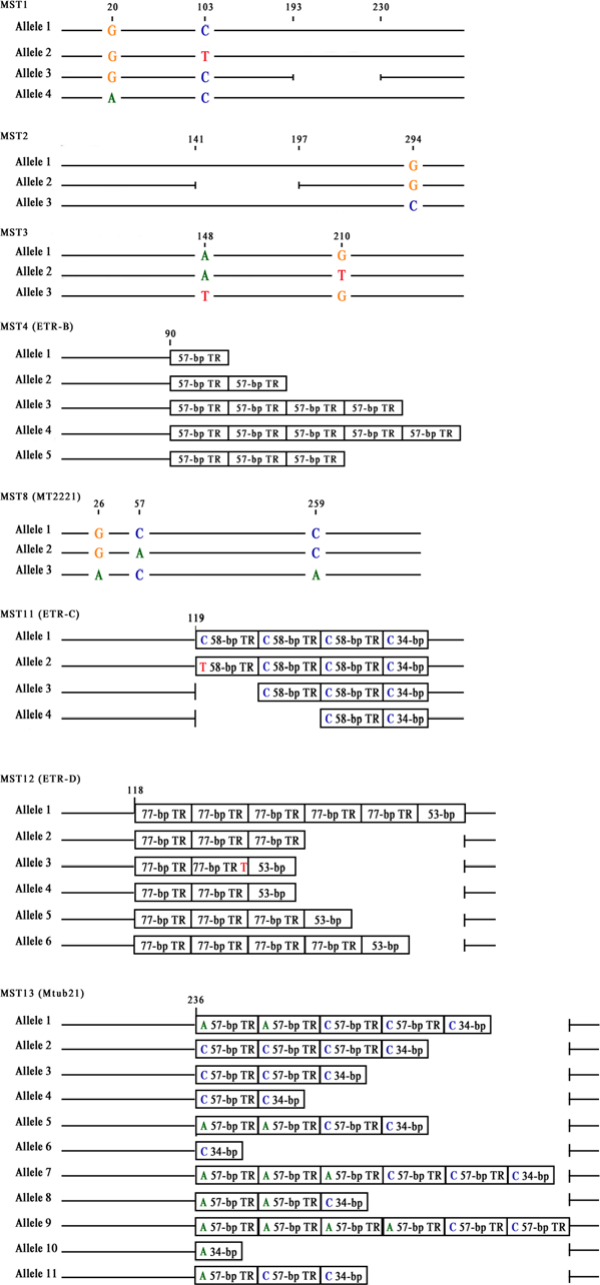
Alleles identified in 8 intergenic spacers in *M. tuberculosis*. Eight intergenic spacers MST1, MST2, MST3, MST4, MST8, MST11, MST12 and MST13 were sequenced in *M. tuberculosis* isolates. Sequences were aligned to highlight differences between isolates, including mutations (colored letters featuring nucleotide bases) and variable number and variable size in tandem repeats (featured by blocks). Numbers in exponent refers to the base position in *M. tuberculosis* H37Rv reference sequence (GenBank: AL123456).

We sequenced the eight selected spacers in a collection of 101 *M. tuberculosis* isolates from our laboratory including the *M. tuberculosis* H37Rv reference strain as well as sequence type (ST) profiles derived *in-silico* from H37Rv and CDC1551 reference strain genomes [29], [30]. Duplicates were carried out for each sequence. Sequence analysis revealed three types of genetic events, i.e. variation in the number of tandem repeat units, single nucleotide mutations and deletions. Of a total of 29 genetic events observed in the eight spacers, 13 (44.82%) were variations in the number of tandem repeats, 11 (37.93%) were single nucleotide mutations and 5 (17.24%) were deletions. As for spacer MST13 (Mtub21), six alleles corresponded to 1–4 copies of a 57-bp repeat unit in addition to an A/C SNP located at the first base of the tandem repeat and a 23-bp deletion (Table 2). MST4 (ETR-B) exhibited five alleles corresponded to 1–5 copies of a 57-bp repeat unit. MST12 (ETR-D) exhibited four alleles including one allele derived from *M. tuberculosis* H37Rv reference strain sequencing. These four alleles combined 2, 3 or 5 copies of a 77-bp repeat unit, a T/G at the position 45 of the second tandem repeat and a 24-bp deletion. MST11 (ETR-C) had four alleles corresponding to 1–3 copies of a 58-bp repeat unit and a T/C SNP at the first base of the tandem repeat in addition to 24-bp deletion. MST1 exhibited four alleles combining mutation A/G at position 20, T/C at position 103 and a 37-bp deletion. MST8 (MT2221) had three alleles combining one A/G mutation at position 26, one A/C mutation at position 57 and one A/C mutation at position 259. MST2 yielded three alleles combining one C/G mutation at position 294 and a 57-bp deletion. MST3 exhibited three alleles due to combining one T/A mutation at position 148, and one T/G mutation at position 210 (Figure 2). Combination of these 32 spacer alleles in the initial collection of 101 *M. tuberculosis* isolates studied yielded 31 different spacer-types (STs) in addition to one profile extracted *in silico* from the *M. tuberculosis* CDC1551 reference strain. The *M. tuberculosis* H37Rv reference strain exhibited the same experimental ST profile as expected from *in silico* prediction (Appendix S1).

MST yielded stable ST profiles when applied to serial *M. tuberculosis* isolates from five different patients. MST profiles were also stable after two subcultures of four different isolates. The discriminatory power of MST typing was calculated to be 0.95. A MST database freely available in our web-site: http://ifr48.timone.univ-mrs.fr/MST_MTuberculosis/mst was built by entering the on-going 32 spacer-type profiles also deposited in GenBank database under accession number (EF202526-EF202555).

The distribution of *M. tuberculosis* isolates into phylogeographical lineages and ST profile was correlated, and 28/31 ST profiles were included into one of the five phylogeographical lineages determined in this study (Figure 1) whereas ST4 was found in both the West-African lineage 1 (two isolates) and the Euro-American lineage (28 isolates); ST5 was found in both West-African lineage 2 (one isolate) and the Euro-American lineage (one isolate) and ST11 was found in both the West-African lineage 1 (two isolates) and West-African lineage 2 (one isolate). The difference between phylogeographical clustering and MST clustering of *M. tuberculosis* isolates was not significant (*P* = 0.2).

### Comparison of MST with reference genotyping methods

Negative controls remained negative in all PCR experiments, and each one of the 93 *M. tuberculosis* DNA under study yielded PCR products of the expected size range. Sequence analyses of the eight spacers identified 737 known sequences and seven new sequences [GenBank: EF559232-EF559238]. Two new ETR-D spacer sequences were observed due to the combination of 3 or 4 copies of 77-bp repeat unit and five new sequences in Mtub21 spacer corresponded to 0, 2, 5 and 6 copies of a 57-bp repeat unit in addition to an A/C single nucleotide polymorphism of the first base of the tandem repeat, and a 23-bp deletion (Figure 2). Combination of spacer sequences yielded 41 different STs including 9 new described STs (Appendix S2); 25 STs were unique and 16 STs clusters comprised of 2 to 12 isolates.

Comparison of MST data with previously determined IS*6110*-RFLP data indicated that 48 *M. tuberculosis* isolates (51.61%) clustered in the same way using MST and IS*6110*-RFLP typing, including 28 isolates clustered in nine MST and 13 IS*6110*-RFLP profiles; and 20 unique isolates. Different clustering was obtained in 45 (48.38%) isolates, including 38 isolates with unique IS*6110*-RFLP profile, clustered in 12 MST profiles comprising of 2–12 isolates, and seven isolates clustered into two IS*6110*-RFLP profiles but typed as unique by MST. The Hunter Gaston index (HGI) was 0.961 for MST and 0.992 for IS*6110*-RFLP (Table 3).

**Table 3:** Discriminatory power of MST compared to RFLP-IS*6110* used alone and in association.

Typing methods	No. of different patterns	No. of clusters	No. of clustered isolates	No of unique isolates	Index Hunter-Gaston (HGI)
MST	41	16	68	25	0.961
RFLP IS*6110*	72	15	35	58	0.992
MST+RFLP IS*6110*	78	13	28	65	0.998

We observed that among the 28 isolates exhibiting < 8 IS*6110* copy number, IS*6110*- RFLP analysis yielded 19 profiles including 14 unique profiles and five clusters of 2–5 isolates, whereas MST yielded 22 profiles including 17 unique STs and five clusters of 2–3 isolates (Table 4). MST was significantly (*P*<0.0003) more discriminatory than IS*6110*-RFLP in the subset of *M. tuberculosis* isolates with less than 8 IS*6110* copies (HGI = 0.969 and 0.917, respectively).

**Table 4:** MST analysis of 33 *M. tuberculosis* isolates exhibiting less than 8 IS*6110* copies.

Isolates	MST1	MST2	MST3	MST4 (ETR-B)°	MST8 (MT2221)	MST11 (ETR-C)	MST12 (ETR-D)	MST13 (Mtub21)	MST profile	*IS6110* Profile	IS*6110*Copy Number
Tub88	1	1	1	2	3	3	4	10	40	IS64	7
Tub94	1	1	1	2	3	3	4	10	40	IS64	7
Tub85	1	1	3	2	3	1	1	5	17	IS54	5
Tub91	1	1	3	2	3	1	1	5	17	IS54	5
Tub1	1	1	1	1	1	1	1	1	1	IS1	2
Tub2	1	1	1	1	1	1	1	1	1	IS1	2
Tub77	1	1	1	1	1	1	1	1	1	IS2	1
Tub90	1	1	1	2	3	4	1	2	7	IS13	4
Tub96	1	1	1	2	3	4	1	2	7	IS13	4
Tub69	1	1	1	3	1	1	5	9	34	IS33	4
Tub70	1	1	1	3	1	1	5	9	34	IS34	2
Tub33	1	1	1	3	1	1	1	1	18	IS2	1
Tub37	1	1	1	1	2	1	1	4	22	IS2	1
Tub46	1	1	1	2	1	1	2	5	25	IS2	1
Tub78	1	1	2	1	1	1	1	3	35	IS2	1
Tub43	1	1	1	3	1	4	5	7	41	IS2	1
Tub25	1	1	2	2	3	3	3	1	13	IS49	7
Tub80	1	1	1	2	3	3	3	10	37	IS63	7
Tub81	1	1	2	5	3	3	5	5	38	IS74	7
Tub42	1	1	1	4	1	4	2	3	24	IS29	4
Tub34	1	3	1	2	3	4	1	5	19	IS22	2
Tub21	1	1	1	2	3	3	3	4	2	IS5	7
Tub14	1	1	2	2	3	3	3	2	5	IS39	7
Tub66	1	1	1	2	3	4	3	4	21	IS26	7
Tub31	1	1	2	2	3	1	3	2	11	IS47	7
Tub67	1	1	1	2	3	3	5	4	33	IS61	6
Tub49	1	1	2	2	3	1	2	4	26	IS56	4
Tub45	1	1	1	2	3	3	3	3	10	IS15	6

**Figure 3:**
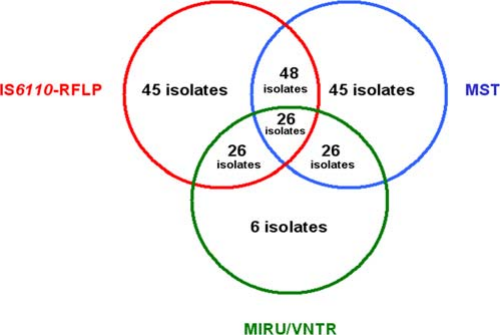
*M. tuberculosis* clustering by using MST, IS*6110*-RFLP and MIRU/VNTR. *M. tuberculosis* isolates were genotyped by using IS*6110*-RFLP (93 isolates, red circle), MIRU-VNTR methods (32 isolates, green circle) and MST (93 isolates, blue circle).

When results were combined, a total of 78 different MST-RFLP-IS*6110* profiles were obtained (Table 3). Sixty-five isolates were unique and 28 isolates grouped into 13 clusters. The HGI for combined methods was of 0.996. Comparison between MST, RFLP-IS*6110* and MIRU/VNTR groupings showed that the five large clusters of the total 41 MST profile, each containing 6 to 12 isolates, could be split by RFLP-IS*6110* typing into nine subclusters concordant with MIRU/VNTR clustering. Five small MST clusters of 1–3 isolates were correctly identified with RFLP-IS*6110* typing and concordant clustering was found with MIRU/VNTR. Finally, all 26 unique MST isolates were correctly identified as unique when subtyped by IS*6110*-RFLP except six single IS*6110* copy isolates which remained grouped into the same IS*6110*-RFLP cluster. These isolates were further found to be unique when analysed with MIRU/VNTR. Altogether, MST, IS*6110*-RFLP and VNTR/MIRU yielded identical results in 26/32 (81.25%) isolates analysed using the three typing methods (*P* =  0.000002) (Figure 3). Discrepant results (18.75%) were due to the six single copy isolates clustering together by IS*6110*-RFLP and were shown to be unique using both MST and MIRU/VNTR typing.

### Clinical specimens

In all PCR experiments, negative controls including non-inoculated mix and 10 respiratory tract specimens remained negative whereas positive amplification was obtained for 7/10 clinical specimens exhibiting Cts ≤42 cycles. Sequencing identified ST12 profile in two clinical specimens and ST5, ST26, ST19, ST8 and ST11 profile in each one of five other clinical specimens. No amplification was obtained for 3/10 clinical specimens exhibiting Cts > 42 cycles in ITS real-time PCR.

## Discussion

Genotyping of 100 representative *M. tuberculosis* clinical isolates and the H37Rv reference strain using the MST approach identified 31 ST profiles and a further ST profile was predicted from the sequence of the *M. tuberculosis* CDC1551 strain analysed *in-silico*. The distribution of these *M. tuberculosis* isolates into ST profiles was significantly correlated with the phylogeographical lineages [13]. The fact that 3 STs did not uniquely match geographical lineage should not be overemphasized at this stage being not statistically significant. This observation warrants further analyses. MST profiling proved reproducible, as stable ST profiles were observed in pairs of *M. tuberculosis* isolates made over 12 to 24 months in five patients with epidemiological and clinical data suggestive of relapsing pulmonary tuberculosis. Also, MST profiles proved stable after two subcultures made from four different *M. tuberculosis* isolates.

MST relies upon sequence-based analysis of eight variable intergenic spacers selected after *M. tuberculosis* genome analysis. Four spacers have been previously described as comprising of VNTR loci corresponding to three ETR loci and the Mtub21 locus [17], [38], [39]. For each spacer, three types of genetic events were observed, i.e. variation in the number of tandem repeats, deletions and point mutations. Latter genetic events have not been described in these loci: VNTR-based methods analyse only the size of amplicons regardless of their sequence [17] and the SNaPshot method [39] relies on hybridation of probes to detect previously know SNPs. Our sequencing approach allowed the identification of three new mutations in addition to the enumeration of tandem repeat copies. These three mutations accounted for 20% of the genetic events in these four spacers. Interestingly, we found that mutation in the first position of the repeat were not always duplicated in the following repeats contrary to that previously reported [40]. Sequencing four additional spacers allowed the identification of eight mutations including one previously described SNP in MT2221 [21] and seven new mutations. Blind comparison between MST and reference IS*6110*-RFLP indicated a 51.61% correlation in the clustering of *M. tuberculosis* isolates. As previously quoted for VNTR/MIRU [41] and spoligotyping [42] the number of IS copies greatly influenced the comparative performance of MST, proved to be more discriminatory than IS*6110*-RFLP in the subset of *M. tuberculosis* isolates exhibiting < 8 IS*6110* copies. *M. tuberculosis* strains widespread in south-east Asia and south Africa contain low copy numbers of the insertion IS*6110* [43], [44] and are not typable using only IS*6110*-RFLP. Indeed, previous observations indicated that IS*6110* clusters with low copy numbers frequently required differentiation by a supplementary technique such as VNTR MIRU typing [43] or spoligotyping [45], [46].

Results from this study indicate that no single method defined all unique isolates; combination of MST with IS*6110*-RFLP achieved the best level of discrimination and therefore increased the probability that clustered isolates were epidemiologically linked.

Previous methods developed for *M. tuberculosis* typing [47], [48] relied upon the analysis of band profiles obtained after genomic restriction as in PFGE or amplification revealed by ethidium bromide staining or IS*6110* probing. Comparison of profiles generated by PFGE may be sometimes difficult in case of poor separation of the different fragments [14], [15]. IS*6110*-RFLP analysis requires large amounts of DNA and is difficult to standardize between laboratories [49]. Discriminatory power of VNTR-based methods depends on the number and set of VNTR loci used; also, there is evidence that the discriminatory power of each loci may vary within each genetic family [50]. Spoligotyping is less discriminatory than IS*6110*-RFLP and not able to discern transmission events especially in regions with predominant or endemic strains [51], [52]. Deletion mapping may require the interpretation of negative results [5]. Deletion analysis is useful for studying the molecular evolution of *M. tuberculosis*, albeit with low discriminatory power. SNP based genotyping has been recently used for *M. tuberculosis* but this analysis does not examine sequence of nucleotide stretches but rather point mutation detected by an hybridization-based method SnaPshot [21]. The MST takes advantage of PCR-based approaches, including requirement for a minimum amount of material, thus limiting the risk of contamination of laboratory workers. We demonstrated that MST could be applied directly on respiratory tract specimens containing *M. tuberculosis* DNA for a rapid molecular epidemiological analysis of tuberculosis. Preliminary results were obtained from clinical specimens exhibiting ≤42 cycles on ITS real-time PCR; further improvements in DNA extraction may allow the application of MST to specimens containing lower inoculum. MST takes advantage of sequencing to yield definitive and exportable data to be immediately compared to other sequences through the use of internet databases without exchanging strains. A free internet MST database has been developed for this purpose (http://ifr48.timone.univ-mrs.fr/MST_MTuberculosis/mst).

In conclusion, we have established the MST approach for the genotyping of *M. tuberculosis* isolates. After validation on a set of *M. tuberculosis* clinical isolates, this method proved to be sensitive, accurate, reproducible and in concordance with phylogeographical lineage assignment. This new approach for *M. tuberculosis* genotyping, requiring minimal *M. tuberculosis* DNA, is particularly adapted to field epidemiology of tuberculosis in tropical countries by exchanging small quantities of DNA or computerised sequence data. Data presented indicate that MST could be used as an alternative to reference methods for the genotyping of *M. tuberculosis* isolates. MST is of particular interest for isolates harbouring less than eight IS*6110* copies and could be applied directly to clinical specimens harbouring acid fast bacilli.

## Supporting Information

Appendix S1 Allele combination of 8 spacers which allow the definition of spacer-types in a collection of 101 M. tuberculosis isolates.
(0.08 MB DOC)

Appendix S2 Genetic analysis of 93 M. tuberculosis isolates using MST and IS6110-RFLP.
(0.26 MB DOC)
