# Supplementary material for: Multispacer Sequence Typing for Mycobacterium tuberculosis Genotyping
Source: PLoS One. 2008 Jun 18;3(6):e2433. doi: 10.1371/journal.pone.0002433 (PMC2413405; doi:10.1371/journal.pone.0002433)
Supplement: Appendix S1 — Allele combination of 8 spacers which allow the definition of spacer-types in a collection of 101 M. tuberculosis isolates. (0.08 MB DOC) [file pone.0002433.s001.doc]

**Appendix S1.** Allele combination of 8 spacers which allow the definition of spacer-types in a collection of 101 *M. tuberculosis* isolates.

| ST |  |  |  | **Spacer combination^*^** | | |  |  |
| --- | --- | --- | --- | --- | --- | --- | --- | --- |
|  |  |  |  |  |  |  |  |  |
|  | MST1 | MST2 | MST3 | MST4 | MST8 | MST11 | MST12 | MST13 |
|  |  |  |  | (ETR-B) | (MT2221) | (ETR-C) | (ETR-D) | (Mtub21) |
| 1 | 1 | 1 | 1 | 2 | 3 | 1 | 4 | 1 |
| 2 | 1 | 1 | 1 | 3 | 1 | 1 | 4 | 6 |
| 3 | 1 | 1 | 1 | 2 | 3 | 1 | 4 | 4 |
| 4 | 1 | 1 | 1 | 2 | 3 | 1 | 4 | 3 |
| 5 | 1 | 1 | 1 | 3 | 1 | 1 | 4 | 1 |
| 6 | 2 | 1 | 2 | 2 | 3 | 3 | 1 | 2 |
| 7 | 1 | 1 | 1 | 3 | 3 | 1 | 1 | 1 |
| 8 | 1 | 1 | 1 | 2 | 3 | 1 | 3 | 2 |
| 9 | 1 | 1 | 1 | 4 | 1 | 1 | 4 | 1 |
| 10 | 1 | 2 | 1 | 2 | 3 | 1 | 4 | 4 |
| 11 | 1 | 1 | 1 | 3 | 1 | 1 | 3 | 4 |
| 12 | 3 | 1 | 1 | 2 | 1 | 1 | 1 | 5 |
| 13 | 1 | 1 | 2 | 2 | 3 | 3 | 3 | 1 |
| 14 | 1 | 1 | 1 | 2 | 3 | 4 | 2 | 5 |
| 15 | 1 | 1 | 1 | 4 | 1 | 4 | 1 | 1 |
| 16 | 1 | 2 | 1 | 2 | 3 | 4 | 2 | 5 |
| 17 | 1 | 1 | 3 | 2 | 3 | 1 | 1 | 5 |
| 18 | 1 | 1 | 1 | 3 | 1 | 1 | 1 | 5 |

| **ST** |  |  |  | **Spacer combination^*^** | | |  |  |
| --- | --- | --- | --- | --- | --- | --- | --- | --- |
|  |  |  |  |  |  |  |  |  |
|  | MST1 | MST2 | MST3 | MST4 | MST8 | MST11 | MST12 | MST13 |
|  |  |  |  | (ETR-B) | (MT2221) | (ETR-C) | (ETR-D) | (Mtub21) |
| 19 | 1 | 3 | 1 | 2 | 3 | 4 | 1 | 5 |
| 20 | 1 | 1 | 2 | 2 | 3 | 4 | 3 | 5 |
| 21 | 1 | 1 | 1 | 2 | 3 | 4 | 3 | 4 |
| 22 | 1 | 1 | 1 | 3 | 1 | 3 | 4 | 5 |
| 23 | 1 | 1 | 1 | 3 | 1 | 2 | 2 | 4 |
| 24 | 1 | 1 | 1 | 4 | 1 | 4 | 2 | 3 |
| 25 | 1 | 1 | 1 | 2 | 1 | 1 | 2 | 5 |
| 26 | 1 | 1 | 2 | 1 | 2 | 1 | 2 | 4 |
| 27 | 1 | 1 | 1 | 3 | 1 | 2 | 4 | 6 |
| 28 | 1 | 1 | 1 | 2 | 3 | 1 | 1 | 3 |
| 29 | 4 | 1 | 1 | 4 | 3 | 2 | 1 | 1 |
| 30 | 1 | 1 | 1 | 2 | 1 | 1 | 1 | 1 |
| 31 | 3 | 1 | 1 | 5 | 3 | 1 | 2 | 6 |
| 32 | 1 | 2 | 1 | 2 | 3 | 3 | 4 | 4 |

^*^: the indexes of allele refer to that described in text and Figure 1, ST31 and ST32 alleles were derived from *M. tuberculosis* H37Rv reference strain and complete genome sequence of *M. tuberculosis* CDC1551 extracted *in silico* (GenBank: AE000516), respectively.
