# Supplementary material for: Multispacer Sequence Typing for Mycobacterium tuberculosis Genotyping
Source: PLoS One. 2008 Jun 18;3(6):e2433. doi: 10.1371/journal.pone.0002433 (PMC2413405; doi:10.1371/journal.pone.0002433)
Supplement: Appendix S2 — Genetic analysis of 93 M. tuberculosis isolates using MST and IS6110-RFLP. (0.26 MB DOC) [file pone.0002433.s002.doc]

**Appendix S2**. Genetic analysis of 93 *M. tuberculosis* isolates using MST and IS*6110*-RFLP.

|  | MST1 | MST2 | MST3 | MST4 | MST8 | MST11 | MST12 | MST13 | MST | *IS6110* | Additional VNTR/ |
| --- | --- | --- | --- | --- | --- | --- | --- | --- | --- | --- | --- |
|  |  |  |  | (ETR-B)° | (MT2221) | (ETR-C) | (ETR-D) | (Mtub21) | profile | Profile | MIRU Profile |
| Tub4 | 1 | 1 | 1 | 2 | 3 | 3 | 3 | 4 | 2 | IS3 | M3 |
| Tub6 | 1 | 1 | 1 | 2 | 3 | 3 | 3 | 4 | 2 | IS3 | M4 |
| Tub73 | 1 | 1 | 1 | 2 | 3 | 3 | 3 | 4 | 2 | IS11 |  |
| Tub89 | 1 | 1 | 1 | 2 | 3 | 3 | 3 | 4 | 2 | IS12 | M5 |
| Tub95 | 1 | 1 | 1 | 2 | 3 | 3 | 3 | 4 | 2 | IS12 | M5 |
| Tub50 | 1 | 1 | 1 | 2 | 3 | 3 | 3 | 4 | 2 | IS7 |  |
| Tub51 | 1 | 1 | 1 | 2 | 3 | 3 | 3 | 4 | 2 | IS9 |  |
| Tub20 | 1 | 1 | 1 | 2 | 3 | 3 | 3 | 4 | 2 | IS4 |  |
| Tub21 | 1 | 1 | 1 | 2 | 3 | 3 | 3 | 4 | 2 | IS5 |  |
| Tub47 | 1 | 1 | 1 | 2 | 3 | 3 | 3 | 4 | 2 | IS6 |  |
| Tub52 | 1 | 1 | 1 | 2 | 3 | 3 | 3 | 4 | 2 | IS8 |  |
| Tub71 | 1 | 1 | 1 | 2 | 3 | 3 | 3 | 4 | 2 | IS10 |  |
| Tub9 | 1 | 1 | 2 | 2 | 3 | 3 | 3 | 2 | 5 | IS38 | M15 |
| Tub10 | 1 | 1 | 2 | 2 | 3 | 3 | 3 | 2 | 5 | IS38 | M15 |
| Tub74 | 1 | 1 | 2 | 2 | 3 | 3 | 3 | 2 | 5 | IS43 |  |
| Tub18 | 1 | 1 | 2 | 2 | 3 | 3 | 3 | 2 | 5 | IS40 | M16 |
| Tub19 | 1 | 1 | 2 | 2 | 3 | 3 | 3 | 2 | 5 | IS40 | M16 |
| Tub38 | 1 | 1 | 2 | 2 | 3 | 3 | 3 | 2 | 5 | IS42 |  |
| Tub76 | 1 | 1 | 2 | 2 | 3 | 3 | 3 | 2 | 5 | IS44 |  |
| Tub26 | 1 | 1 | 2 | 2 | 3 | 3 | 3 | 2 | 5 | IS41 | M17 |
| Tub48 | 1 | 1 | 2 | 2 | 3 | 3 | 3 | 2 | 5 | IS41 | M17 |
| Tub14 | 1 | 1 | 2 | 2 | 3 | 3 | 3 | 2 | 5 | IS39 |  |
| Tub58 | 1 | 1 | 1 | 2 | 3 | 4 | 2 | 5 | 14 | IS17 | M7 |
| Tub61 | 1 | 1 | 1 | 2 | 3 | 4 | 2 | 5 | 14 | IS17 | M7 |
| Tub62 | 1 | 1 | 1 | 2 | 3 | 4 | 2 | 5 | 14 | IS17 | M7 |
| Tub63 | 1 | 1 | 1 | 2 | 3 | 4 | 2 | 5 | 14 | IS17 | M7 |
| Tub75 | 1 | 1 | 1 | 2 | 3 | 4 | 2 | 5 | 14 | IS19 |  |
| Tub72 | 1 | 1 | 1 | 2 | 3 | 4 | 2 | 5 | 14 | IS18 |  |
| Tub44 | 1 | 1 | 1 | 2 | 3 | 4 | 2 | 5 | 14 | IS16 |  |
| Tub30 | 1 | 1 | 3 | 2 | 3 | 1 | 1 | 5 | 17 | IS52 |  |
| Tub86 | 1 | 1 | 3 | 2 | 3 | 1 | 1 | 5 | 17 | IS55 |  |
| Tub92 | 1 | 1 | 3 | 2 | 3 | 1 | 1 | 5 | 17 | IS55 |  |
| Tub85 | 1 | 1 | 3 | 2 | 3 | 1 | 1 | 5 | 17 | IS54 | M18 |
| Tub91 | 1 | 1 | 3 | 2 | 3 | 1 | 1 | 5 | 17 | IS54 | M18 |
| Tub60 | 1 | 1 | 3 | 2 | 3 | 1 | 1 | 5 | 17 | IS53 |  |
|  | MST1 | MST2 | MST3 | MST4 | MST8 | MST11 | MST12 | MST13 | MST | *IS6110* | Additional VNTR/ |
|  |  |  |  | (ETR-B)° | (MT2221) | (ETR-C) | (ETR-D) | (Mtub21) | profile | Profile | MIRU Profile |
| Tub87 | 1 | 1 | 1 | 2 | 3 | 1 | 2 | 3 | 4 | IS37 | M14 |
| Tub93 | 1 | 1 | 1 | 2 | 3 | 1 | 2 | 3 | 4 | IS37 | M14 |
| Tub13 | 1 | 1 | 1 | 2 | 3 | 1 | 2 | 3 | 4 | IS66 |  |
| Tub8 | 1 | 1 | 1 | 2 | 3 | 1 | 2 | 3 | 4 | IS65 |  |
| Tub22 | 1 | 1 | 1 | 2 | 3 | 1 | 2 | 3 | 4 | IS67 |  |
| Tub68 | 1 | 1 | 1 | 2 | 3 | 1 | 2 | 3 | 4 | IS68 |  |
| Tub1 | 1 | 1 | 1 | 1 | 1 | 1 | 1 | 1 | 1 | IS1 | M1 |
| Tub2 | 1 | 1 | 1 | 1 | 1 | 1 | 1 | 1 | 1 | IS1 | M1 |
| Tub77 | 1 | 1 | 1 | 1 | 1 | 1 | 1 | 1 | 1 | IS2 | M2 |
| Tub90 | 1 | 1 | 1 | 2 | 3 | 4 | 1 | 2 | 7 | IS13 | M6 |
| Tub96 | 1 | 1 | 1 | 2 | 3 | 4 | 1 | 2 | 7 | IS13 | M6 |
| Tub12 | 1 | 1 | 1 | 2 | 3 | 4 | 1 | 2 | 7 | IS69 |  |
| Tub36 | 1 | 1 | 1 | 2 | 3 | 4 | 3 | 4 | 21 | IS25 | M9 |
| Tub65 | 1 | 1 | 1 | 2 | 3 | 4 | 3 | 4 | 21 | IS25 | M9 |
| Tub66 | 1 | 1 | 1 | 2 | 3 | 4 | 3 | 4 | 21 | IS26 |  |
| Tub31 | 1 | 1 | 2 | 2 | 3 | 1 | 3 | 2 | 11 | IS47 |  |
| Tub32 | 1 | 1 | 2 | 2 | 3 | 1 | 3 | 2 | 11 | IS48 |  |
| Tub23 | 1 | 1 | 2 | 2 | 3 | 1 | 3 | 2 | 11 | IS70 |  |
| Tub88 | 1 | 1 | 1 | 2 | 3 | 3 | 4 | 10 | 40 | IS64 | M19 |
| Tub94 | 1 | 1 | 1 | 2 | 3 | 3 | 4 | 10 | 40 | IS64 | M19 |
| Tub35 | 1 | 1 | 2 | 2 | 3 | 4 | 3 | 5 | 20 | IS23 |  |
| Tub41 | 1 | 1 | 2 | 2 | 3 | 4 | 3 | 5 | 20 | IS24 |  |
| Tub69 | 1 | 1 | 1 | 3 | 1 | 1 | 5 | 9 | 34 | IS33 |  |
| Tub70 | 1 | 1 | 1 | 3 | 1 | 1 | 5 | 9 | 34 | IS34 |  |
| Tub67 | 1 | 1 | 1 | 2 | 3 | 3 | 5 | 4 | 33 | IS61 |  |
| Tub84 | 1 | 1 | 1 | 2 | 3 | 3 | 5 | 4 | 33 | IS62 |  |
| Tub49 | 1 | 1 | 2 | 2 | 3 | 1 | 2 | 4 | 26 | IS56 |  |
| Tub82 | 1 | 1 | 2 | 2 | 3 | 1 | 2 | 4 | 26 | IS57 |  |
| Tub27 | 1 | 1 | 1 | 4 | 3 | 4 | 2 | 3 | 15 | IS20 |  |
| Tub28 | 1 | 1 | 1 | 4 | 3 | 4 | 2 | 3 | 15 | IS50 |  |
| Tub45 | 1 | 1 | 1 | 2 | 3 | 3 | 3 | 3 | 10 | IS15 |  |
| Tub17 | 1 | 1 | 1 | 2 | 3 | 3 | 3 | 3 | 10 | IS40 | M16 |
| Tub33 | 1 | 1 | 1 | 3 | 1 | 1 | 1 | 1 | 18 | IS2 | M8 |
| Tub37 | 1 | 1 | 1 | 1 | 2 | 1 | 1 | 4 | 22 | IS2 | M10 |
| Tub46 | 1 | 1 | 1 | 2 | 1 | 1 | 2 | 5 | 25 | IS2 | M11 |
| Tub78 | 1 | 1 | 2 | 1 | 1 | 1 | 1 | 3 | 35 | IS2 | M12 |

| Isolates | MST1 | MST2 | MST3 | MST4 | MST8 | MST11 | MST12 | MST13 | MST | *IS6110* | Additional VNTR/ |
| --- | --- | --- | --- | --- | --- | --- | --- | --- | --- | --- | --- |
|  |  |  |  | (ETR-B)° | (MT2221) | (ETR-C) | (ETR-D) | (Mtub21) | profile | Profile | MIRU Profile |
| Tub43 | 1 | 1 | 1 | 3 | 1 | 4 | 5 | 7 | 41 | IS2 | M13 |
| Tub16 | 1 | 1 | 1 | 1 | 3 | 1 | 3 | 2 | 9 | IS14 |  |
| Tub54 | 1 | 1 | 1 | 2 | 3 | 1 | 1 | 3 | 28 | IS59 |  |
| Tub83 | 3 | 1 | 1 | 5 | 3 | 1 | 5 | 10 | 39 | IS75 |  |
| Tub25 | 1 | 1 | 2 | 2 | 3 | 3 | 3 | 1 | 13 | IS49 |  |
| Tub80 | 1 | 1 | 1 | 2 | 3 | 3 | 3 | 10 | 37 | IS63 |  |
| Tub81 | 1 | 1 | 2 | 5 | 3 | 3 | 5 | 5 | 38 | IS74 |  |
| Tub64 | 1 | 2 | 1 | 2 | 3 | 3 | 4 | 4 | 32 | IS60 |  |
| Tub55 | 4 | 1 | 1 | 4 | 3 | 2 | 1 | 1 | 29 | IS31 |  |
| Tub53 | 1 | 1 | 1 | 2 | 3 | 1 | 1 | 2 | 27 | IS58 |  |
| Tub59 | 3 | 1 | 1 | 5 | 3 | 1 | 2 | 6 | 31 | IS32 |  |
| Tub7 | 1 | 1 | 1 | 2 | 3 | 1 | 3 | 5 | 3 | IS36 |  |
| Tub79 | 1 | 1 | 2 | 2 | 3 | 1 | 1 | 10 | 36 | IS73 |  |
| Tub24 | 3 | 1 | 1 | 2 | 1 | 1 | 1 | 5 | 12 | IS71 |  |
| Tub11 | 2 | 1 | 2 | 2 | 3 | 3 | 1 | 2 | 6 | IS45 |  |
| Tub15 | 1 | 1 | 1 | 2 | 3 | 1 | 3 | 2 | 8 | IS46 |  |
| Tub42 | 1 | 1 | 1 | 4 | 1 | 4 | 2 | 3 | 24 | IS29 |  |
| Tub39 | 1 | 1 | 1 | 3 | 1 | 2 | 2 | 4 | 23 | IS28 |  |
| Tub34 | 1 | 3 | 1 | 2 | 3 | 4 | 1 | 5 | 19 | IS22 |  |
| Tub56 | 1 | 1 | 1 | 2 | 1 | 1 | 1 | 1 | 30 | IS72 |  |
| Tub29 | 1 | 2 | 1 | 2 | 3 | 4 | 2 | 5 | 16 | IS51 |  |
| Tub 40 | 1 | 1 | 3 | 2 | 3 | 1 | 1 | 5 | 17 | IS24 |  |
